# Effects of Increased Standing and Light-Intensity Physical Activity to Improve Postprandial Glucose in Sedentary Office Workers: Protocol for a Randomized Crossover Trial

**DOI:** 10.2196/45133

**Published:** 2023-08-23

**Authors:** Shannon L Wilson, Rachel Crosley-Lyons, Jordan Junk, Kristina Hasanaj, Miranda L Larouche, Kevin Hollingshead, Haiwei Gu, Corrie Whisner, Dorothy D Sears, Matthew P Buman

**Affiliations:** 1 College of Health Solutions Arizona State University Phoenix, AZ United States; 2 Keck School of Medicine University of Southern California Los Angeles, CA United States; 3 College of Health Sciences Midwestern University Glendale, AZ United States; 4 Center for Translational Science Florida International University Port St. Lucie, FL United States

**Keywords:** sitting bouts, digital health intervention, standing desk, endothelial function, glycemic control, sleep, blood pressure, insulin, mHealth

## Abstract

**Background:**

Prolonged bouts of sedentary time, independent from the time spent in engaging in physical activity, significantly increases cardiometabolic risk. Nonetheless, the modern workforce spends large, uninterrupted portions of the day seated at a desk. Previous research suggests—via improved cardiometabolic biomarkers—that this risk might be attenuated by simply disrupting sedentary time with brief breaks of standing or moving. However, this evidence is derived from acute, highly controlled laboratory experiments and thus has low external validity.

**Objective:**

This study aims to investigate if similar or prolonged cardiometabolic changes are observed after a prolonged (2-week) practice of increased brief standing and moving behaviors in real-world office settings.

**Methods:**

This randomized crossover trial, called the WorkWell Study, will compare the efficacy of two 2-week pilot intervention conditions designed to interrupt sitting time in sedentary office workers (N=15) to a control condition. The intervention conditions use a novel smartphone app to deliver real-time prompts to increase standing (STAND) or moving (MOVE) by an additional 6 minutes each hour during work. Our primary aim is to assess intervention-associated improvements to daily postprandial glucose using continuous glucose monitors. Our secondary aim is to determine whether the interventions successfully evoke substantive positional changes and light-intensity physical activity (LPA). Other outcomes include the feasibility and acceptability of the intervention conditions, fasting blood glucose concentration, femoral artery flow-mediated dilation (f-FMD), and systolic and diastolic blood pressure.

**Results:**

The trial is ongoing at the time of submission.

**Conclusions:**

This study is a novel, randomized crossover trial designed to extend a laboratory-based controlled study design into the free-living environment. By using digital health technologies to monitor and prompt participants in real time, we will be able to rigorously test the effects of breaking up sedentary behavior over a longer period of time than is seen in traditional laboratory-based studies. Our innovative approach will leverage the strengths of highly controlled laboratory and free-living experiments to achieve maximal internal and external validity. The research team’s multidisciplinary expertise allows for a broad range of biological measures to be sampled, providing robust results that will extend knowledge of both the acute and chronic real-life effects of increased standing and LPA in sedentary office workers. The WorkWell Study uses a rigorous transdisciplinary protocol that will contribute to a more comprehensive picture of the beneficial effects of breaking up sitting behavior.

**Trial Registration:**

ClinicalTrials.gov NCT04269070; https://clinicaltrials.gov/study/NCT04269070

**International Registered Report Identifier (IRRID):**

DERR1-10.2196/45133

## Introduction

Society is rapidly evolving to embrace the technological advances in communication, entertainment, and transportation that have benefited nearly every sector of daily life. These advances have also contributed to the accumulation of time spent engaging in sedentary behaviors, with approximately 60% of waking hours spent sedentary [1]. Sedentary behavior (defined by the Sedentary Behavior Research Network as <1.5 metabolic equivalents in a seated posture) [2] is a growing public health concern due to its well-established links to cardiometabolic disease development and premature mortality, independently from time spent in moderate to vigorous physical activity (MVPA) [3-7]. Meeting the recommended 150 minutes per week of MVPA is associated with beneficial cardiometabolic health outcomes [8-11]. However, MVPA alone does not offset the numerous negative health outcomes associated with prolonged bouts of inactivity. Research suggests that for each additional hour spent sedentary, the likelihood of developing metabolic syndrome and type 2 diabetes increases by 39% and 22%, respectively [12]. Therefore, reducing time spent in prolonged bouts of inactivity may lead to favorable long-term health outcomes [13].

Breaking up prolonged bouts of sitting attenuates the negative health outcomes associated with sedentary behavior [14-20], likely due to postural change and contraction of large skeletal muscle groups [13]. These actions result in increased vascular blood flow and shear stress via gravitational pull and the skeletal muscle pump, thus increasing venous return, decreasing blood pooling in the lower limbs, and activating contraction- and insulin-mediated glucose uptake pathways [21]. Taking a break to stand, walk, or exercise—in some cases, for as little as 2 minutes for every 20 minutes spent sitting [14,17]—improves glycemic control [14-19,22], insulin sensitivity [16,17], vascular response [14,23], and cardiometabolic health outcomes [16,19].

Adults spend 7.7 to 11.5 hours per day engaging in sedentary time [4,24,25], with modern office-based employees spending more than 89% of their workday seated [20]. This sedentary time, however, might be reduced through various intervention techniques. A meta-analysis reported that environmental (eg, sit-stand workstation at work) and multicomponent (eg, sit-stand workstation, education, and mobile app) intervention approaches resulted in sedentary time reductions of 40.6 and 35.5 minutes per day, whereas behavioral interventions (eg, education) resulted in a modest reduction of 23.87 minutes of sedentary time [26]. Studies using isotemporal modeling indicate that replacing sitting bouts of 30-60 minutes with either light-intensity physical activity (LPA) or MVPA is associated with reduced risk for all-cause and cardiovascular mortality, and improved cardiometabolic risk biomarkers, particularly in adults who are less active [27,28]. These findings support the feasibility and efficacy of interventions targeting sedentary time reduction and resulting health benefits [26-31].

Intervention-based research aimed at reducing sitting time and improving health outcomes has typically been conducted in tightly controlled laboratory environments, and the few studies conducted in real-world settings lack the intensive clinical and behavioral measures of a laboratory-based study. Thus, workplace-based efficacy and feasibility studies incorporating rigorous procedures with high internal validity are needed to better evaluate interventions aimed at reducing sitting time. The protocol described is a workplace study using evidence-based intervention components along with mobile and wearable technologies. A randomized crossover design will be used to explore cardiometabolic outcomes resulting from the addition of brief periods of standing or LPA during work hours while also assessing the efficacy and feasibility of the intervention in the real-world office setting.

## Methods

### Study Aims

The primary aim of this study is to examine the impact of 2 different intervention conditions (STAND and MOVE) on 3-hour postlunch postprandial glucose compared to the baseline control condition. Interventions entail adding 6 minutes per hour to baseline standing and LPA minutes during the workday. We hypothesize that both intervention conditions will improve postprandial glucose regulation. The secondary aim is to assess intervention adherence and changes in standing and LPA. Exploratory aims include (1) assessing participant acceptability and satisfaction during the intervention; and assessing intervention-induced changes in (2) sleep, (3) anthropometrics and body composition, (4) endothelial function, (5) central and peripheral blood pressure, (6) fasting blood glucose concentration, (7) fasting insulin concentration, (8) fasting high-density lipoprotein (HDL) concentration, (9) low-density lipoprotein (LDL) oxidation, (10) fasting triglycerides, (11) total antioxidant capacity (TAC), (12) hemoglobin A_1c_ (HbA_1c_), (13) microbial diversity of the gut, (14) metabolomics from stool and plasma, and (15) DNA variants that may explain differences in response to the intervention.

### Study Design

**Figure 1:**
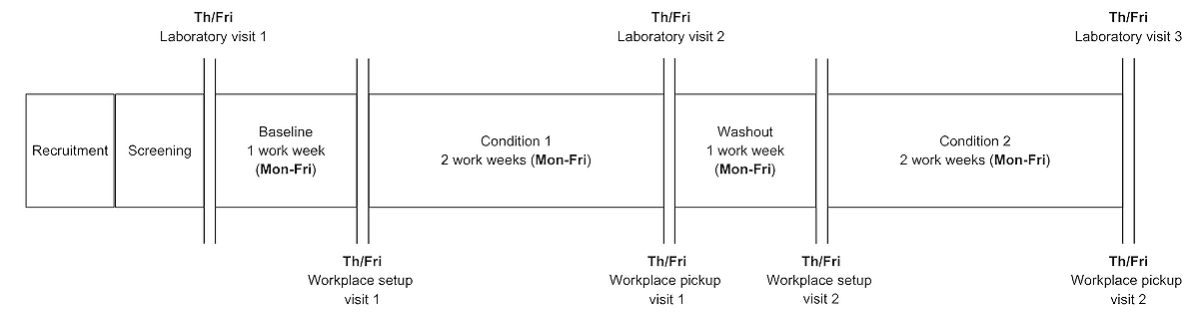
WorkWell Study timeline.

The WorkWell Study is a pilot 6-week randomized crossover design with 2 balanced arms. Across 2 intervention periods, all participants (N=15) will experience both workplace interventions following a baseline control condition: (1) MOVE, an intervention targeting 6-minute increase in baseline LPA each work hour and (2) STAND, an intervention targeting 6-minute increase in baseline standing each work hour via a sit-stand workstation. Participants will wear a Bluetooth accelerometer and will be prompted with a mobile app during each intervention period. The intervention sequence is randomized and a washout period of at least 1 week will occur between interventions to reduce any potential carryover effect. Clinic-based assessments will occur at baseline and after each intervention period is completed (Figure 1).

### Study Eligibility

Potential participants will go through a biphasic screening process to determine eligibility, see full eligibility criteria in Textbox 1. Participants will complete the web-based screening questionnaire, which will be reviewed by staff to determine initial eligibility; individuals who are conditionally eligible will be asked to consent to continue the screening process and to complete a medical history review. Next, an in-laboratory visit will confirm BMI eligibility. Those with qualifying BMIs are considered eligible to participate in the WorkWell Study and will have the opportunity to undergo full informed consent.

**Eligibility criteria**

**Inclusion criteria**

- Age of ≥18 years
- Primary work activities performed seated
- Ability to use sit-stand workstation
- Works ≥4 days per week (in-office or remote)
- BMI of ≥25 (≥23 for Asian descent)

**Exclusion criteria**

- Taking certain medications and supplements, which includes glucose control, blood pressure, and blood thinner medications; hormone replacement therapy; corticosteroids; high-dose statins; second-generation antipsychotics; and prebiotics, probiotics, or antibiotics (in previous 3 months)
- Inability to stand or move for prolonged periods due to lower limb injury, disability, preexisting medical condition, or doctor’s orders
- Serious medical conditions including, neuromuscular, cardiovascular, and respiratory disorders, as well as inflammatory bowel or intestinal malabsorption conditions, or food allergies
- Actively using a sit-stand workstation
- Work travel of ≥3 days per week
- Smoking

### Procedures

#### Study Recruitment

Participants will be recruited on a rolling basis from the Arizona State University community via email listserves, slack channels, e-newsletters, staff website advertisements, and word-of-mouth. Preexisting partner businesses and organizations throughout the Phoenix Metropolitan area will be contacted by email. Employee-facing flyers containing study information and a link to the screening questionnaire will be disseminated electronically through the aforementioned channels.

#### Baseline Period

Once enrolled and initial baseline measures taken, participants will go through a 1-week baseline period in which they are outfitted with multiple devices that they will wear during each intervention period: a continuous glucose monitor (FreeStyle Libre, Abbott Diabetes Care), activPAL micro (PAL Technologies), and GENEActiv accelerometer (ActivInsights). These devices will be worn continuously (ie, 24 hours per day) for 7 consecutive days to assess baseline glycemic response, activities (including sitting, standing, and lying behaviors), and sleep, respectively. Participants will engage in their normal behaviors during the week-long baseline period.

Lunch will be delivered on workdays during the baseline period and both intervention periods. Participants will select options from a standardized menu (approximately 33% of daily caloric needs is equal to 660 kcals, with macronutrient composition of 55% carbohydrate is equal to approximately 90.75 g, 30% fat carbohydrate is equal to approximately 22 g, 15% protein [±10%] is equal to 24.75 g) created from a list of local vendors. The standardized meal choices will be consistently repeated each week during the trial, with the same meals provided Monday to Friday during baseline and both intervention periods. Participants will be asked to avoid eating or drinking (with the exception of water and black coffee or tea) 3 hours before and post lunchtime. Food logs will be used to report when they started and finished eating their lunch, how much food they ate, and the exact food consumed during the trial.

#### Randomization

After completion of the baseline period, each participant will be randomized to an intervention sequence (MOVE-STAND or STAND-MOVE). Randomization is completed using the REDCap (Research Electronic Data Capture; Vanderbilt University) randomization tool and a preloaded table with treatment sequences [32,33].

#### Interventions

**Figure 2:**
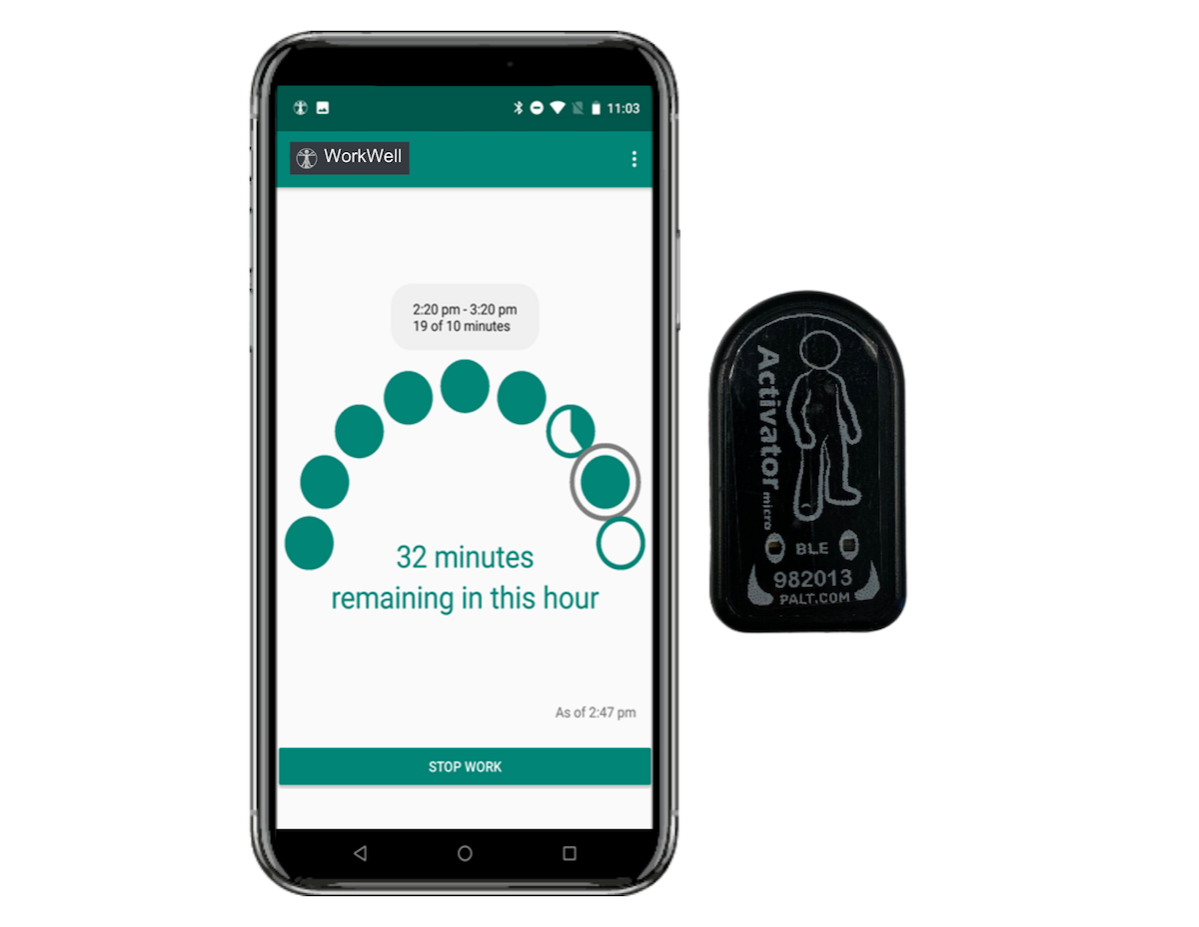
WorkWell intervention tools—WorkWell mobile app and Activator micro device.

The MOVE and STAND interventions will be delivered across 2 work weeks (approximately 10 days). Both use the WorkWell mobile app and Bluetooth Activator micro (PAL Technologies) device as intervention tools in conjunction with the equipment worn during the baseline period (Figure 2). The primary behavioral target of the MOVE intervention will be to increase baseline LPA time by an additional 6 minutes per hour during work. Similarly, the primary behavioral target of the STAND intervention will be to increase baseline standing time by an additional 6 minutes per hour during work. This target of adding 6 minutes per working hour of standing and LPA was selected as an achievable goal with evidence for cardiometabolic health improvements [14-19,22].

After the baseline period is completed, the self-reported work hours and corresponding activPAL micro data will be used to identify the average minutes of standing and moving for each hour of the participant’s workday. The intervention goals will be identified for each participant by adding 6 minutes to these hourly averages. For example, if a participant stands for an average of 10 minutes per work hour during the baseline period, their STAND intervention goal will be 16 minutes of standing per work hour. The same approach will be used to identify MOVE intervention goals. The WorkWell app will use live feedback from the Bluetooth Activator micro to prompt participants in the STAND intervention to meet their hourly standing goals using a sit-stand workstation, while the MOVE intervention will prompt participants to engage in light-intensity movements (eg, walking) to meet their hourly goals.

Prior to the start of the intervention, staff will meet with participants to provide the devices, equipment, and logs needed during the intervention period. Participants will undergo an introductory tour before each intervention and are reminded of their intervention goal for that period. During the intervention, participants will wear the Bluetooth Activator micro device on the mid-thigh and use the WorkWell mobile app during their workday in conjunction with the continuously-worn continuous glucose monitoring (CGM), activPAL, and GENEActiv. When the workday begins, participants will wear the Bluetooth Activator micro and log their work start time in the app, signaling the “start” of the intervention for that day. The Activator micro device will send real time accelerometer data to the WorkWell app via a Bluetooth signal, providing live participant-facing feedback on hourly goal progress as well as day-level summary information. Summary data will also be sent to a secure server, allowing study staff to monitor participant compliance and to facilitate support when needed. Participants will additionally receive a variety of prompts, including hourly reminders to work toward their hourly goal, encouraging messages, or messages that acknowledge that they achieved their hourly goal. Participants will be instructed to log the end of their workday in the app before removing the Bluetooth Activator micro, thus signaling the “end” of that day's intervention.

#### Measures

All measurements will be completed at the baseline visit and after each intervention period, for a total of 3 laboratory visits. Table 1 shows the measures collected and the associated measurement period. Demographic and health history information will be assessed at baseline. During the baseline period, as well as during each intervention period, participants will complete 3 paper-form daily logs for sleep, work, and food recall. All other study data and questionnaires will be collected and managed using REDCap hosted at Arizona State University [32,33]. REDCap is a secure, web-based software platform designed to support data capture for research studies, providing (1) an intuitive interface for validated data capture, (2) audit trails for tracking data manipulation and export procedures, (3) automated export procedures for seamless data download to common statistical packages, and (4) procedures for data integration and interoperability with external sources.

**Table 1:** WorkWell measurement schedule.

Measures				Baseline	Intervention 1	Intervention 2
**Screening**						
		Demographics and health history		✓^a^	—^b^	—
		BMI (kg/m^2^)		✓^c^	—	—
**Primary outcomes**						
		Postprandial blood glucose (incremental area under the curve)		✓^d^	✓^d^	✓^d^
**Secondary outcomes**						
		Intervention adherence (% goal met)		✓^d^	✓^d^	✓^d^
		Posture/activity (minutes/day)		✓^d^	✓^d^	✓^d^
**Exploratory outcomes**						
	Sleep			✓^d^	✓^d^	✓^d^
	Intervention feasibility			✓^a^	✓^a^	✓^a^
	**Anthropometrics**					
			Body weight (kg)	✓^c^	✓^c^	✓^c^
			Body fat percentage (%)	✓^c^	✓^c^	✓^c^
			Waist circumference (cm)	✓^c^	✓^c^	✓^c^
	Endothelial function (% flow-mediated dilation)			✓^c^	✓^c^	✓^c^
	Blood pressure (mm Hg)			✓^c^	✓^c^	✓^c^
	**Fasted blood measures**					
			Glucose (mg/dL)	✓^c^	✓^c^	✓^c^
			Insulin (μlU/mL)	✓^c^	✓^c^	✓^c^
			HDL-cholesterol (mg/dL)	✓^c^	✓^c^	✓^c^
			LDL oxidation (U/L)	✓^c^	✓^c^	✓^c^
			Triglycerides (mg/dL)	✓^c^	✓^c^	✓^c^
			Total antioxidant capacity (µmol/L)	✓^c^	✓^c^	✓^c^
	Microbial diversity			✓^c^	✓^c^	✓^c^
	Hemoglobin A_1c_ (mg/dL)			✓^c^	N/A^e^	N/A
	Metabolomics (stool and plasma)			—	—	—
	DNA variants			✓^c^	—	—

^a^Self-administered survey via REDCap (Research Electronic Data Capture).

^b^Not measured.

^c^Measured at each laboratory visit.

^d^Measured continuously (device-based).

^e^N/A: not applicable.

#### Primary Outcomes

Our primary outcome, the change in 3-hour postlunch postprandial glucose excursion, will be assessed using a CGM device. This small, single-use sensor is placed on the back of the arm and samples glucose levels approximately once every 15 minutes for up to 14 days [34]. This device provides a valid and reliable measure of glucose using flash glucose monitoring [35,36]. Research staff will facilitate the insertion of the CGM for baseline and both intervention periods. If the device falls off or is accidentally removed, staff will replace the device as needed. Participants will be provided a macronutrient-controlled meal (approximately 33% of daily caloric needs, with macronutrient composition of 55% carbohydrate, 30% fat, 15% protein [±10%]) delivered daily for lunch during the work week to control for dietary variability. The 3-hour postlunch postprandial incremental area under the curve (iAUC) and mean blood glucose concentration in response to the lunchtime meal will be calculated from these data.

#### Secondary Outcomes

The secondary outcomes of the interventions will assess intervention adherence and changes in sitting, standing, and LPA during work hours. Intervention adherence is defined as meeting the hourly intervention goal of ≥80% of the workday (eg, meeting the hourly goal for ≥8 hours during a 10-hour workday). The Activator micro device will be taped midthigh during work hours and will communicate with the WorkWell smartphone app via Bluetooth technology. The app’s user-friendly interface will provide real time feedback, allowing participants to monitor their progress as 8 bubbles gradually turn green to signify the hourly goals that were met. The WorkWell app will sync to a secure server, providing real time stand-and-move data for study staff to monitor adherence to intervention protocols; data will be stored for broader analysis upon trial conclusion.

The activPAL micro will also assess sitting, standing, and moving behaviors. Work logs will be used to differentiate between work and nonwork hours. Device attachment procedures are based on several large-scale intervention studies [5,6,37]. Devices will be waterproofed using a medical grade adhesive covering and placed on the midline of the thigh with breathable, hypoallergenic tape, to allow for 24 hours per day of continuous wear during measurement periods without removing for bathing or other water-based activities. Data will be processed into bouts of sitting or lying, standing, stepping, or time in bed (ie, primary lying bout) and expressed as minutes per day, assessing posture and intensity using activPAL software version 8.10. Waking time assessed as lying or seated will be categorized as sedentary behavior. Stepping time will be split into periods of LPA (<100 steps per minute) and MVPA (≥100 steps per minute) [38]. Total physical activity will be a cumulative daily value of LPA and MVPA. The following periods of wear will be excluded: (1) continuous sitting or standing behavior >6 hours (considered nonwear), (2) days with ≤10 hours of valid wear time during the wake period, and (3) participants with only 1 valid day of activPAL wear.

#### Exploratory Outcomes

Additional exploratory outcomes will include sleep, intervention acceptability and satisfaction, body anthropometrics, femoral artery flow-mediated dilation (f-FMD), central aortic blood pressure, fasting glucose, fasting insulin, HDL, LDL, LDL oxidation, triglycerides, TAC, gut microbial diversity, HbA_1c_, metabolomics of the blood and stool, and genetic variants assessed during 1 or more measurement periods.

Sleep will be assessed using the wrist-worn GENEActiv accelerometer (ActivInsights) (initialized to collect at 40 Hz); participants will wear the device 24 hours per day during the measurement periods while also completing a validated sleep diary each night to identify sleep from wake periods. The GENEActiv device has been validated for sleep [39-42] and will be used in conjunction with a sleep log that differentiates sleep or wake periods in data [43]. An open-source and validated algorithm (GGIR package, R-software) [42] will be used to process device-based sleep measures, including sleep duration, sleep efficiency, sleep onset latency, and wakefulness after sleep onset [44-47]. Sleep will be considered the total sleep time accumulated within the sleep window; wake time, daytime sleep, and the time of sleep onset will not be counted. GGIR signal processing includes autocalibration using local gravity as a reference [45], detection of prolonged abnormal high values, nonwear detection and average magnitude of dynamic acceleration calculation, corrected for average gravity over 5-second epochs and reported in milligravitational units (mg). Data files will be excluded if a postcalibration error of >0.01 g (10 mg), <3 days of valid wear (defined as >16 hours per day [44,48]), or wear data are not present for each 15 minutes period of the 24-hour cycle. Nonwear detection is described elsewhere [44]. The default nonwear setting will be used, which includes invalid data that will be imputed by the average at similar time points on different days during the assessment week. Sleep duration will be calculated using automated sleep detection (HDCZA sleep detection algorithm) [47]. The average of all valid days will be used.

Intervention acceptability and satisfaction will be assessed using the Treatment Evaluation Questionnaire (TEQ) to measure the adherence and feasibility outcomes of the study. Prior to starting each intervention, participants will be provided an introductory tour to explain the intervention and describe how to use the WorkWell app and device. Research staff will actively discuss strategies to assist participants in engaging with the intervention. After this session and prior to the start of the intervention, participants will be sent a pre-study survey with the TEQ; once each intervention is completed, a postsurvey is sent also containing the TEQ.

Body anthropometrics, including height, weight, body fat percentage, and waist circumference, will be measured at each laboratory visit. Height and weight will be measured using the Seca scale and stadiometer (Seca 286 EMR Ready Ultrasonic Measuring Station). Waist circumference (taken at the level of the iliac crest) will be measured twice using a Gulick tape measure. Body fat percentage will be measured twice using the Tanita TBF-400 digital scale device (Tanita), which calculates percent body fat using bioimpedance. If there is a difference of ≥0.2% for body fat, an additional measurement will be taken. Values will be averaged to arrive at a final measure.

f-FMD will be used to measure the endothelium-dependent, flow-mediated dilation of the superficial femoral artery using B-mode ultrasound (Terason uSmart 3300+TM) via guidelines set forth by the Brachial Artery Reactivity Task Force [49]. Participants will be asked to lie quietly for 15 minutes on the ultrasound table before baseline images are obtained from their left upper thigh. Simultaneous ultrasound images (B-mode) and Doppler waveforms will be recorded for 30 seconds. An appropriately sized cuff will be selected based on manufacturer guidelines (Hokanson Instruments) and subsequently wrapped around the participant’s upper thigh, 3-4 cm below the bifurcation of the common femoral artery. After baseline images of the superficial femoral artery are acquired, the blood pressure cuff on the participant’s upper thigh will be inflated using a rapid-cuff inflator (Hokanson instruments) to a suprasystolic pressure of 250 mm Hg for 5 minutes. The recording of the image will begin 30 seconds prior to the cuff release. At 5 minutes following cuff inflation, the cuff will be rapidly deflated and arterial images are recorded for 6 minutes. Images obtained will be analyzed by a trained, blinded researcher using previously validated edge-detection software [50]. In our laboratory, intraclass correlation coefficients for baseline and peak diameter are 0.994 and 0.995, respectively (Cronbach α=.976).

Central aortic blood pressure that includes both central and peripheral blood pressure, and aortic stiffness (via applanation tonometry) will be measured using the SphygmoCor XCEL (CvMS; ATCOR Medical). This device has been found to reflect measures of central pressures and stiffness reliably and accurately [51,52]. All measurements will be taken after the participant rests in a supine position for approximately 15 minutes, prior to f-FMD initiation, to ensure hemodynamic stability. Triplicate measures will be collected, and the 2 closest values averaged and reported.

Fasting blood samples will be collected at each laboratory visit via venipuncture. The 15-mL samples will be immediately processed and aliquoted for storage at –80 °C for subsequent analysis. Fasting blood glucose will be examined in multiple batches, pairing samples from the same participant’s previously aliquoted plasma. Plasma glucose concentration will be analyzed using the AU480 Chemistry Analyzer (Beckman Coulter, Inc) in accordance with manufacturer’s instructions. Exploratory cardiometabolic disease risk analysis will be performed on plasma and serum glucose, insulin, triglycerides, total cholesterol, HDL cholesterol, LDL cholesterol, LDL oxidation, and TAC. Exploratory, targeted metabolomics analyses will also be performed with plasma samples using mass spectrometry (MS).

Participants will be provided with a stool sample collection kit and instructions at each laboratory visit. All participants will be given the option to schedule sample pick-up at their home or workplace; alternatively, samples can be dropped off at the Translational Research Center. Participants will be instructed to collect this sample within 4 days of each laboratory visit for a total of 3 collections. Once collected, samples will be frozen at –80 °C within 24 hours of the sample leaving the body. Participants will be instructed to contact staff to retrieve the sample when ready. The stool samples will later be thawed at 4 °C and microbial DNA extracted for microbiome sequencing and characterization of intervention-specific bacterial community differences. Microbial DNA sequencing will be carried out in batches of paired samples from the same participant. Bacterial DNA will be extracted from the stool samples using DNeasy PowerSoil Pro DNA Isolation Kit (47014, Qiagen) as per manufacturer recommendations. DNA quality and concentration will be quantified (QIAxpert System Qiagen) before being sent out for sequencing (Illumina MiSeq instrument, Illumina, Inc.) at ASU’s DNASU Genomics Core Facility. Raw Illumina microbial data will be cleaned and analyzed using the Quantitative Insights into Microbial Ecology 2 software, as previously described [53]. Exploratory, targeted metabolomics analyses will be performed with the stool samples using MS. Short-chain fatty acids, bile acids, pathway-specific targeted liquid chromatography tandem MS, liquid chromatography–MS, gas chromatography–MS aqueous metabolite profiling, and liquid chromatography–MS global lipidomic profiling will be carried out.

HbA_1c_ will be collected at baseline only using the DCA Vantage analyzer (Siemens Munich) with 1 μL of blood obtained using a finger stick. After collection, the results are obtained within 6 minutes and data will be used for descriptive purposes.

Saliva samples will be collected at baseline using Oragene Discover collection kits (OGR-600 Ottawa), and will be archived for future exploration of genetic variants.

### Data Analysis

#### Statistical Analysis Plan

Participant characteristics will be reported as frequencies or means with SDs. Outcome variables with nonnormal distributions will be log-transformed to assume normal distribution. All data processing and statistical analyses will be performed in SAS (version 9.4; SAS Institute) with an α criterion of .05. To address our specific aims and hypotheses, we will use linear mixed model analyses [54,55] for intervention (ie, MOVE and STAND), sequence (ie, MS and SM), and time period (ie, 1 or 2) as fixed factors and an unstructured covariance structure for the 3 repeated measurements per person. The primary outcome will be postprandial glucose iAUC, and secondary outcome will be change in posture and LPA. All data from randomized participants will be included in the analyses in accordance with intent-to-treat principles.

#### Sample Size Calculation

To determine the sample size needed, we based our effect size estimates on previous studies [17,56] that have similarly evaluated the impact of interrupting prolonged sitting on glucose iAUC. These studies reported a 20%-30% decrease in iAUC level for the intervention groups. Therefore, for the WorkWell Study, we used a conservative estimate of 15% difference between baseline and the 2 intervention measurements (with a 1% population estimate of SD). Using G*Power software (version 3.1.9.2) [57], we estimated that we will have to recruit 15 individuals (allowing for 80% retention) to obtain a final sample size of 12 participants (α=.05, power=80%).

### Ethics Approval

This study has been reviewed and approved by the institutional review board at Arizona State University (STUDY00010172) as well as registered on ClinicalTrials.gov (NCT04269070). Screening consent will be obtained prior to in-person screening visit and subsequent full-informed consent will be obtained from all qualifying participants prior to enrollment. All participant information and data will be deidentified and assigned an identification number in which all subsequent data, samples, and surveys will be labeled to ensure confidentiality. As a part of the study intervention, participants will be provided lunchtime meals during the work week as well as up to US $65 in a web-based gift card of their choice for completion of the study.

## Results

Recruitment is ongoing as of January 2023. To date, 10 participants have been enrolled, with 2 withdrawing from the study for personal reasons. Enrollment was completed in March 2023.

## Discussion

### Comparison to Prior Work

While low levels of physical activity have been a public health concern, there is evidence that sedentary behavior duration may also confer health risks [13]. Observational studies suggest that breaking up sedentary time is associated with improved cardiometabolic health [58]. These free-living, mainly observational studies have demonstrated the theoretical feasibility of workplace interventions, yet cannot control for potentially confounding factors, obscuring the meaningful analysis of the relationship. Laboratory studies, however, suggest a causal relationship between decreased sedentary time and cardiometabolic health [59]. While these studies are tightly controlled in both the environment and intervention, they lack important context regarding the translation of these findings into real-world settings.

The WorkWell Study is a novel randomized controlled crossover trial that has been designed to extend laboratory-based, controlled study designs into the natural environment of the workplace. To accomplish this, we have used digital health technologies to monitor and prompt participants in real time while maintaining rigorous adherence to increased standing and moving regimes. This innovation will allow us to test the effects of breaking up sitting with standing and LPA over a longer period of intervention than has been used in traditional laboratory-based studies. Additionally, we have included a broad set of concomitant biological measures, including dynamic changes in postprandial and 24-hour glucose, as well as outcomes in the emerging areas of metabolome and gut microbiome composition. We will use two 2-week interventions targeting behavior change to decrease sitting time in the workplace as well as to improve cardiometabolic health and to assess behavior change. This study was carefully designed to extend laboratory-based studies—both in duration, as well as translation into more ecological settings—thus providing more evidence on the causal role of interrupted sitting time (with standing and moving) on cardiometabolic health. As a result, we will be able to address (1) statistical association via baseline and washout weeks that allow participants to serve as their own controls, (2) temporality via continuous accelerometer tracking and prescheduled laboratory visits, and (3) nonspuriousness via randomized assignment and controlled treatment dosage.

### Strengths and Limitations

Our project attempts to achieve levels of internal validity previously found only in laboratory-based studies while simultaneously maximizing external validity. While others have demonstrated the benefit of breaking up sedentary behavior in either highly controlled laboratory environments or ecological environments, we will use emerging technologies (ie, wearable sensors and smartphone apps) to deliver a rigorous clinical trial within an employee's real-life workspace. Despite this important strength, some potential challenges warrant discussion. Recruitment and retention of participants in longitudinal research is challenging. We hope to overcome this challenge by allowing participants to engage in the study in their typical work setting, rather than having participants carry out the intervention in a laboratory setting. If successful, this approach will add important information to our current understanding of how best to intervene and engage participants in reducing sedentary time in workplace settings. Participants may also feel uneasy about collecting fecal samples. To ensure this is not a barrier for successful completion of the trial, the study team will provide participants with a discrete collection kit that can be used mostly anywhere that is comfortable for the participant. Delivery of samples to the research facility can then be initiated by the participant or the study staff can retrieve the sample at a place of convenience for the participant. This method has been used by our investigative team in previous studies and has proven beneficial for ensuring receipt of quality samples within the required time frame.

With a wide range of expertise represented on our research team, we are able to consider outcomes in the behavioral, physiological, and microbial realms and are able to examine our outcomes in an integrated and transdisciplinary fashion. While previous research has broadly examined some of these factors, our design and transdisciplinary approach will allow for a more global viewpoint of the influence of breaking up sedentary time during the workday on cardiometabolic health. Currently, the biological and metabolic underpinnings of sedentary behavior-linked disease are grossly understudied. Previous observational work has highlighted potential microbial and metabolic derangements [60-63], but these findings have not been extended to longitudinal interventions, leaving the previous work highly correlational. Our team is well-positioned to advance this area of study toward better mechanistic understandings of sedentary behavior and how intervention in real-world settings can improve health.

### Conclusions

The WorkWell Study is a randomized controlled crossover trial examining the feasibility and impact of breaking up sedentary behavior on cardiometabolic health using the scientific rigor of a laboratory-based study with the context and translatability of a free-living setting. We will explore the effects that standing or moving 6 additional minutes per hour has on postprandial glucose and other physiological measures in the real-world workplace and assess intervention feasibility. Using a rigorous transdisciplinary approach, we seek to fill gaps in previous research and contribute to a more comprehensive picture of the beneficial effects of breaking up sitting behavior.

## Abbreviations

CGM: continuous glucose monitoring
f-FMD: femoral artery flow-mediated dilation
HbA_1c_: hemoglobin A_1c_
HDL: high-density lipoprotein
iAUC: incremental area under the curve
LDL: low-density lipoprotein
LPA: light-intensity physical activity
MS: mass spectrometry
MVPA: moderate to vigorous physical activity
REDCap: Research Electronic Data Capture
TAC: total antioxidant capacity
TEQ: Treatment Evaluation Questionnaire

## Data Availability

The data generated are not publicly available due to the fact that this trial is ongoing. Data will be available from the corresponding author upon reasonable request.
